# Field rice panicle detection and counting based on deep learning

**DOI:** 10.3389/fpls.2022.966495

**Published:** 2022-08-12

**Authors:** Xinyi Wang, Wanneng Yang, Qiucheng Lv, Chenglong Huang, Xiuying Liang, Guoxing Chen, Lizhong Xiong, Lingfeng Duan

**Affiliations:** National Key Laboratory of Crop Genetic Improvement, Agricultural Bioinformatics Key Laboratory of Hubei Province, National Center of Plant Gene Research, College of Engineering, Huazhong Agricultural University, Wuhan, China

**Keywords:** field rice, panicle detection, panicle counting, large image size, deep learning

## Abstract

Panicle number is directly related to rice yield, so panicle detection and counting has always been one of the most important scientific research topics. Panicle counting is a challenging task due to many factors such as high density, high occlusion, and large variation in size, shape, posture et.al. Deep learning provides state-of-the-art performance in object detection and counting. Generally, the large images need to be resized to fit for the video memory. However, small panicles would be missed if the image size of the original field rice image is extremely large. In this paper, we proposed a rice panicle detection and counting method based on deep learning which was especially designed for detecting rice panicles in rice field images with large image size. Different object detectors were compared and YOLOv5 was selected with MAPE of 3.44% and accuracy of 92.77%. Specifically, we proposed a new method for removing repeated detections and proved that the method outperformed the existing NMS methods. The proposed method was proved to be robust and accurate for counting panicles in field rice images of different illumination, rice accessions, and image input size. Also, the proposed method performed well on UAV images. In addition, an open-access and user-friendly web portal was developed for rice researchers to use the proposed method conveniently.

## Introduction

Rice is one of the important cereal crops in the world, especially in Asia. The yield of cereal crops is related to the number of panicles per square meter, grains per panicle and grain size (Slafer et al., 2014; Lu et al., 2015; Ferrante et al., 2017; Jin et al., 2017). Thus, in order to predict the yield of rice, panicle count is an appropriate method. However, manual counting has the defects of high labor cost, time consuming and error-prone. Also, for yield prediction, studies tend to process infield large size images (Fiorani and Schurr, 2013). It is necessary to develop a method to count panicles fast, accurately and automatically for filed images.

**FIGURE 1:**
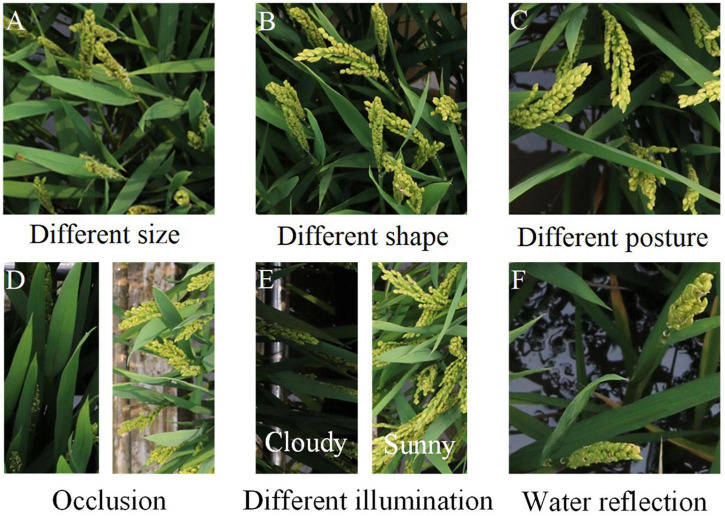
Challenges in rice panicle detection. **(A)** Different size. **(B)** Different shape. **(C)** Different posture. **(D)** Occlusion. **(E)** Different illumination. **(F)** Water reflection.

However, automatic panicle count is an enormous challenge. For panicle detection, complexity of the field environment can bring many difficulties, which is shown in Figure 1, such as different size, different shape, different posture, serious occlusion, different illumination and water refection. With the development of artificial intelligence and machine vision technology, many studies used machines to count the number of fruits for crop yield prediction, such as cotton (Singh et al., 2021), corn (Khaki et al., 2021), sugar-beet (Barreto et al., 2021), citrus (Dorj et al., 2017) and so on. For counting, the current studies for cereal panicle count can be mainly divided into three categories: image segmentation, object detection and counting directly through regressing network.

Image segmentation segments the panicles based on the phenotypic characteristics, such as color and texture. Combined with the counting method, the number of panicles can be counted. Xiong et al. (2017) proposed an algorithm to segment panicles based on superpixel regions generation, CNN and superpixel optimization and the F-measure was 76.73%. Hayat et al. (2020) proposed an algorithm for rice panicle segmentation based on unsupervised Bayesian learning and the mean F1 score was 82.10%. Ma et al. (2020) proposed EarSegNet based on semantic segmentation for winter wheat ears segmentation and the F1 score was 87.25%. Yang et al. (2020) used FPN-Mask model to segment panicles during grain filling stage and the pixel accuracy was 0.99. Misra et al. (2020) proposed SpikeSegNet for wheat spike detection and counting and the average accuracy for spike counting was 95%. Wang et al. (2020) proposed an algorithm using 3D point cloud to obtain agricultural crop dimensions, which was suitable for panicle count at high density. However, this method was designed for indoor images and could not be directly generalized to field. Besides panicle number, panicle shape, size, position and color et al. can also be obtained after panicle segmentation, which is convenient for further phenotypic analysis. However, the accuracy of the counting is largely dependent on the accuracy of panicle segmentation. When the rice panicles occluded with each other, it is hard to separate the panicles. And panicle segmentation needs to be combined with the counting method to obtain the panicle number, which would lead to error accumulation.

Object detection is a common method for counting by detecting and drawing bounding boxes. Besides panicle number, object detection can also obtain information about panicle size and position. Ji et al. (2021) proposed a detection method, which contained light saturation correction and Itti saliency-based system for candidate areas detection and combined with feature extraction and the usage of LS-SVM classifier for elimination of false. The F1 score of this method was 88.36%. However, without deep learning, this method might be limited for directly used in other applications due to comparably insufficient learning ability. In the research of object detection using deep learning, some of the studies directly resized the images due to the need of the deep learning networks. Zhou et al. (2019) proposed an improved R-FCN for rice panicle detection and the F-measure was 87.4%. Yang et al. (2021) proposed an improved YOLOv4 for detection of wheat spikes and the accuracy of the wheat spikes with different density distributions was 94%, 96.04% and 93.11%. However, the above two algorithms directly resized the images before feeding to the model, which might lead to lots of missing of the small panicles as the size of the small panicles would be largely decreased or even disappeared after resize when the original image size was large.

Using object detection for counting panicles in images with large image size, sliding window and image cutting are two commonly used methods. However, repeated detections between the adjacent sub-images bring new challenges. Desai et al. (2019) used a sliding window to detect the flowering regions based deep learning. However, this method counted the regions containing panicles to predict the panicle number, which was not suitable for the situation of dense growth and different sizes of rice panicles. Xu et al. (2020) proposed an algorithm namely multi-scale hybrid window panicle detect (MHW-PD) for rice panicle count. For images with large number of panicles, this algorithm cut the images into sub-images without overlapping and detected the sub-images based on convolutional neural network. If the two bounding boxes in the adjacent sub-images were close and the sum of the area of the two boxes was close to the average size of a panicle, the two boxes would be merged. This algorithm was not suitable for the panicles with different sizes. In addition, the author mentioned that for more dense and occluded rice panicles, the accuracy of the method was reduced and it would cause more miss-detection. For images with 71-80 panicles, the counting accuracy of this algorithm was 86.7%. Lyu et al. (2021) also split the large size images into small tiles and used the DBSCAN algorithm to remove the repeated detections. The average error of this counting method was 33.98%.

Counting directly through regressing network was another commonly used method for object counting. Lu et al. (2017) proposed a regressing network, TasselNet to count tassels directly. However, this method might be less robust in the later growth stage than object detector (Madec et al., 2019). TasselNetV2 and TasselNetV2 + was subsequently proposed by the same research group to improve the counting accuracy and efficiency (Xiong et al., 2019; Lu and Cao, 2020). Compared with other deep convolution neural networks, TasselNetV2 + reduced the use of the video memory and would be able to analyze large size images efficiently. Similarly, Khaki et al. (2022) proposed WheatNet for wheat head counting and its overall prediction error was 8.7%. One disadvantage of the counting directly through regressing network method was that this method can only obtain the panicle number. Thus, it was difficult to make a more specific analysis of the phenotype of panicles after counting.

**FIGURE 2:**
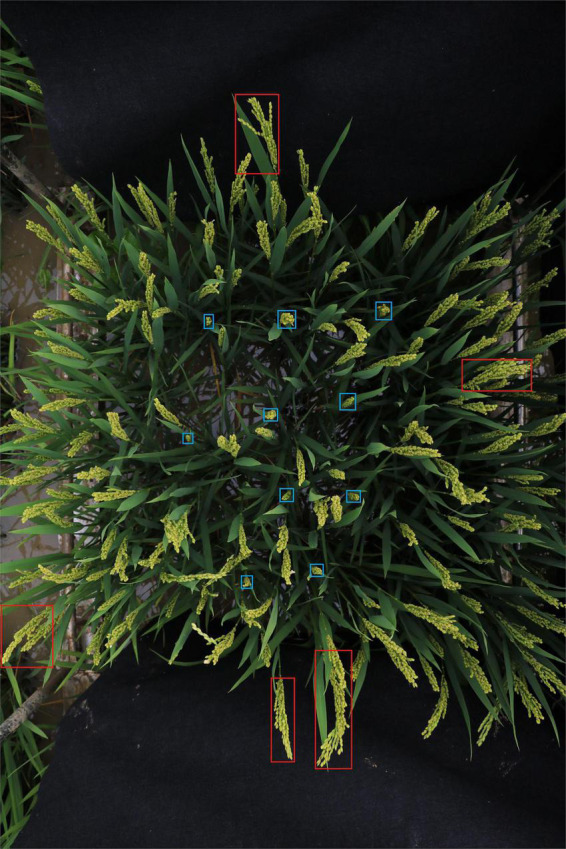
Example of rice field image. The size of rice panicles varies greatly. Red boxes show examples of large panicles and blue boxes show examples of small panicles.

The size of panicles varies greatly even in the same plot. Some panicles would be extremely small (for instance, blue boxes in Figure 2). If the original large size image was directly resized before feeding to the detection network, small panicles would be missed in detection.

To detect and count panicles in rice field images with large image size, an algorithm based on deep learning was proposed in this paper. Firstly, an original high-resolution image was cut into several sub-images in an overlapping manner to ensure that a panicle would be appeared completely in at least one sub-image. Then, the sub-images were fed into the panicle detection networks and the detection results were merged to get the detection result. Three object detectors, namely YOLOv3, YOLOv5 and Faster R-CNN, were used and compared in this study. The repeated detections in the overlapping area of adjacent sub-images were then removed using two indicators. To validate the proposed algorithm, panicle detection for field rice images taken by ground-based imaging system with different illumination, rice accessions and spatial resolution were tested. To further investigate the robustness of the proposed method, panicle detection for field rice images taken by UAV was also tested.

## Materials and methods

### Rice cultivation and image acquisition

In this study, the experimental paddy field was located in Wuhan, Hubei province, China (30.5N, 114.3E). Rice (O. sativa) seeds were sown and germinated during the summer of 2017. Each field plot (96 × 80 cm^2^) had 20 rice plants of the same accessions, which were planted in 5 rows and 4 columns. The spacing between each plant was 16 × 16 cm^2^ and the spacing between each plot was 32 cm. Considered the edge effect, a guard row of rice plants was planted on the boundary between two adjacent plots. Rice plants in different plots belonged to different accessions. In total, 104 rice accessions were used for training and testing in this work. All these accessions come from core germplasm resources of Japonica rice in China. The names of the 104 rice accessions are listed in Supplementary Table 1. The panicle number of each field plot varied from 75 to 190. For each plot, the top-view image was acquired. A ground-based imaging bracket was used to obtain rice plot images. The camera (Canon EOS 760D, 18 mm focal length lens, 6000 × 4000 pixels) were mounted at the top of the imaging bracket. Wireless shutter was used to trigger the camera to take images when the imaging bracket moved manually in the paddy field.

### Main flow of rice panicle detection algorithm

**FIGURE 3:**
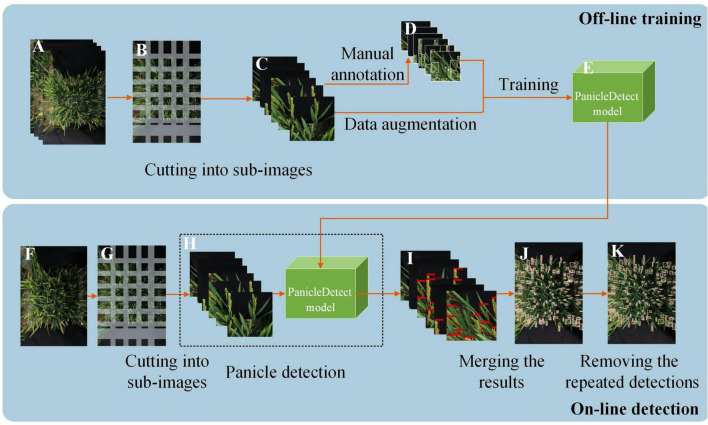
The flow diagram of the panicle detection algorithm. **(A)** Original high-resolution image. **(B)** Cutting into sub-images in an overlapping manner. **(C)** Sub-images. **(D)** Manual annotation and data augmentation. **(E)** Panicle detection model generation. **(F)** Testing sample. **(G)** Cutting the testing sample into sub-images in an overlapping manner. **(H)** Feeding the testing sub-images into the Panicle detection model. **(I)** Panicle detection results of the sub-images. **(J)** Panicle detection result after merging the detection results of the sub-images. **(K)** The final detection results of the original image after deleting the repeated detections in the overlapping area of the adjacent sub-images.

The rice panicle detection algorithm included off-line training and on-line detection (Figure 3).

In total, 104 field-rice images (each image belonged to a different accession) with a resolution of 6000 × 4000 was used for training and testing our panicle detection algorithm. The 104 images were randomly split into 2 sets: 67 images for training and 37 images for testing.

The off-line training mainly contained 3 steps: (1) The original training images were divided into sub-images of appropriate size using sliding windows in an overlapping manner (Figure 3C). (2) The sub-images were annotated using the software, LableImg (Figure 3D); (3) The data was augmented and the PanicleDetect model was trained (Figure 3E).

**FIGURE 4:**
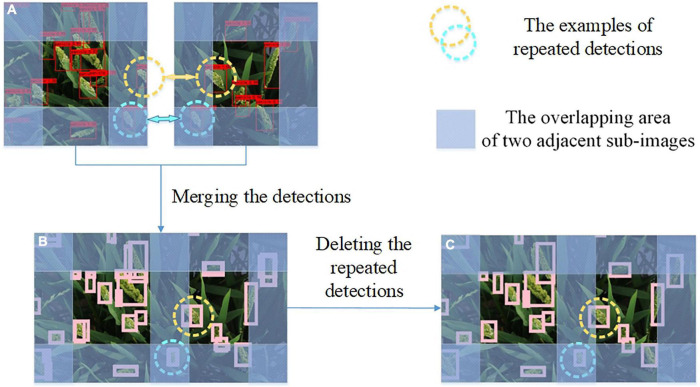
The specific processing steps of deleting the repeated detections. **(A)** Repeated detections in the overlapping area of two adjacent sub-images. **(B)** Merging results directly. **(C)** Deleting the repeated detections in two adjacent sub-images.

The on-line detection stage mainly included 4 steps: (1) An original testing image was divided into sub-images of appropriate size using sliding windows in an overlapping manner (Figure 3C); (2) All sub-images corresponding to the original image were fed into the pre-trained PanicleDetect model (Figure 3H); (3) The detection results of the sub-images (Figure 3I) were merged (Figure 3J); (4) Repeated detections in the overlapping areas of the adjacent sub-images were deleted (Figure 3K).

An open-access and user-friendly web portal^1^ was developed for rice researchers to use the proposed method conveniently. The detailed operation of the website is illustrated in Supplementary Video 1. Users can upload a single image or multiple images at a time. Detection results including the resultant images and a text file recording the panicle number at each image can be downloaded.

### Training of the PanicleDetect model

From the collected 104 images, 67 images were randomly selected and each original image were divided into sub-images using sliding windows in an overlapping manner. The overlapping size (stride) was determined by the average size of the large panicles to ensure that most of the panicles appeared completely in at least one sub-image. And the size of the sub-image was determined by the panicle size, the selected network and the video memory. In this study, the size of sub-image was set as 1056 × 1056 and the stride was set as 756. Therefore, each field rice image was divided into 40 sub-images. In total, 67 × 40 = 2680 sub-images were obtained for training the PanicleDetect model. Then we randomly selected 2144 images for training and 536 images for validation from the 2680 sub-images in an 8:2 ratio.

The PanicleDetect model was built based on YOLOv5x. YOLOv5x is a fully convoluted network. In the structure of backbone of YOLOv5x, the input image needs to be down-sampled for 5 times, and each down-sampling reduces the image size by half. Therefore, the input image size should be a multiple of 32. In this study, all the sub-images were resized to 416 × 416 pixels before feeding to YOLOv5x.

During training of the object detector, the data was augmented using image resizing, image blurring, image flipping and rotating, and transformation of hue, saturation and value. The training was run on the Windows 10 operating system (16-core i7 CPU, 2.5 GHz per CPU core, 16GB of memory, and an NVIDIA GeForce RTX 2070 super graphics card). The network was pre-trained on the COCO-Train2017 dataset, and the generated weight file was loaded as the initial weight. SGD optimizer (Song et al., 2013) was used in the training and the momentum (He et al., 2019) was set to 0.937. The training of model was divided into two stages, each of which trained for 50 epochs. At the first stage, the parameters of the backbone of YOLOv5x were frozen. And at the second stage, all the parameters of YOLOv5x were trained.

### Removing the repeated detections in the overlapping areas

**FIGURE 5:**
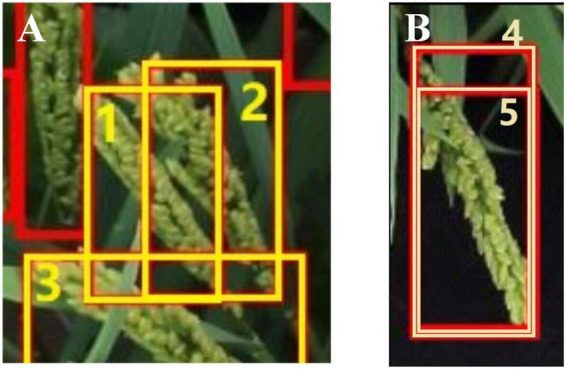
Examples of two overlapping situations. **(A)** high density panicles (1,2,3) that should be retained. **(B)** Repeated detection (4,5) that should be deleted.

The specific processing steps of removing the repeated detections are illustrated in Figure 4. There were two types of overlapping boxes: overlapped panicles that should be retained (Figure 5A) and repeated detections that should be removed (Figure 5B).

Non-maximum suppression (NMS) method using Intersection over Union (IOU) was the most widely used method to quantify and remove the overlapping detection boxes. Furthermore, methods similar to NMS for removing overlapping results have also been proposed, such as GIOU (Rezatofighi et al., 2019) and DIOU (Zheng et al., 2019). The definitions of IOU, GIOU and DIOU are provided in Eqs. (1)–(3).


(1) $$I\,O\,U=\frac{B\,o\,x_{s\,m\,a\,l\,l\,e\,r}\,\cap B\,o\,x_{b\,i\,g\,g\,e\,r}}{B\,o\,x_{s\,m\,a\,l\,l\,e\,r}\,\cup B\,o\,x_{b\,i\,g\,g\,e\,r}}$$

(2) $$G\,I\,O\,U=I\,O\,U-\frac{\left|A_{c}-B\,o\,x_{s\,m\,a\,l\,l\,e\,r}\,\cup B\,o\,x_{b\,i\,g\,g\,e\,r}\right|}{\left|A_{c}\right|}$$

(3) $$D\,I\,O\,U=I\,O\,U-\frac{\rho^{2}}{c^{2}}$$

where A_*c*_ is the smallest enclosing box area, ρ is the Euclidean distance between the center points of two boxes, and c is the diagonal length of the smallest enclosing box.

When the areas of the overlapping boxes were similar, NMS methods can remove the repeated boxes correctly. However, when the areas of the overlapping boxes varied greatly, the union between overlapping boxes was very close to the bigger box, so some NMS methods may not work. In this work, the original large size rice image was divided into small sub-images in an overlapping manner. A panicle would appear in several sub-images. And a small part of a panicle may appear in one sub-image while the complete panicle appears in another sub-image. Therefore, the areas of the overlapping boxes of the repeated detections would vary greatly. In this case, the NMS methods may not be suitable. To remove the repeated detections while retain the overlapped panicles, two parameters, namely IOB and BOU (defined by Equation 4 and 5), were introduced in this manuscript to quantify the overlapping mode and degree between two overlapping boxes. If IOB > threshold of IOB and BOU > threshold of BOU, the bounding box which had a smaller area in two overlapping boxes was removed.


(4) $$I\,O\,B=\frac{B\,o\,x_{s\,m\,a\,l\,l\,e\,r}\,\cap B\,o\,x_{b\,i\,g\,g\,e\,r}}{B\,o\,x_{s\,m\,a\,l\,l\,e\,r}}$$

(5) $$B\,O\,U=\frac{B\,o\,x_{b\,i\,g\,g\,e\,r}}{B\,o\,x_{s\,m\,a\,l\,l\,e\,r}\,\cup B\,o\,x_{b\,i\,g\,g\,e\,r}}$$

where Box_*smaller*_ is the box with the smaller area of the two overlapping boxes and Box_*bigger*_ is the box with the bigger area of the two overlapping boxes.

### Performance evaluation using 6 indicators

In order to test the detection algorithm, 37 rice field images were selected. Six indicators, including the mean absolute percentage error (MAPE), Precision, Recall, F-measured, coefficient of determination (R^2^) and Accuracy were adopted to evaluate the performance of the detection. Among them, MAPE and Accuracy were used to evaluate the detection accuracy of the algorithm. The lower the MAPE and the higher the Accuracy, the more accurate the detection is.

Precision represents that how many panicles detected by the algorithm are ground-truth annotations. And Recall illustrates that among all the panicles identified by the human experts, how many panicles are detected by the algorithm. In practice, Precision and Recall interact with each other, so we need to balance these two indicators. F-measure was used to evaluate the detection performance in a more comprehensive way. A high F-measure value means that the rice panicle detection algorithm has a good performance. In addition, the coefficient of determination (R^2^) was used to test the fitting degree of machine counting results versus manual counting results. The definition of MAPE, Precision, Recall, F-measure and Accuracy are provided in Eqs. (6)–(10).


(6) $$MAPE=\frac{\left(\sum_{i=1}^{n}\frac{\left|y_{i}-{\hat{y}}_{i}\right|}{y_{i}}\right)}{n}\times 100\left(\%\right)$$

(7) $$Precision=\frac{T\,P}{T\,P+F\,P}\times 100\left(\%\right)$$

(8) $$Recall=\frac{T\,P}{T\,P+F\,N}\times 100\left(\%\right)$$

(9) $$F=\frac{2\times P\,r\,e\,c\,i\,s\,i\,o\,n\times R\,e\,c\,a\,l\,l}{P\,r\,e\,c\,i\,s\,i\,o\,n+R\,e\,c\,a\,l\,l}\times 100\left(\%\right)$$

(10) $$Accuracy=\frac{T\,P+T\,N}{T\,P+T\,N+F\,P+F\,N}\times 100\left(\%\right)$$

Where n is the number of the test images, y_*i*_ is the panicle number calculated manually, and y_*i*_ is the panicle number calculated by our algorithm. TP, TN, FP, and FN represent the numbers of true positive, true negative, false positive, and false negative, respectively. In this paper, the true positive (TP) is the number of bounding boxes which detect the rice panicles correctly. The true negative (TN) is always considered to be zero because background is not determined for object detection in this study. The false positive (FP) is the number of bounding boxes which detected backgrounds falsely as rice panicles. The false negative (FN) is the number of ground truth rice panicles which are not detected by the algorithm.

### Robustness evaluation of the PanicleDetect model

To evaluate the robustness of the PanicleDetect model to different rice accessions and illumination, 37 field rice images belonging to 37 different rice accessions were tested for panicle detection. In addition, the illumination of the different images varied due to the outdoor environment.

In order to improve the robustness of the model, the height and width of the input images were randomly scaled in data augmentation at the training stage. To evaluate the robustness of the model to different image size and spatial resolution, the sub-images (1056 × 1056) of the test images were resized to 256 × 256, 416 × 416, 608 × 608, 800 × 800 and 1056 × 1056, respectively, in the testing stage. Subsequently, the sub-images were detected by the model trained with sub-images of 416 × 416 pixels.

To further investigate the universality of the proposed method, panicle detection for field rice images taken by UAV was also tested. The tested UAV images were taken by the camera (FUJIFILM GFX 100 camera, 63 mm focal length lens) installed on the UAV platform (DJI M600 Pro, 20 m flight altitude, 1 m/s flight speed). Before detecting, the height and width of the test images were magnified 3.5 times because of the huge differences in spatial resolution between the UAV images and the training set. The spatial resolution of the UAV images was about 2mm per pixel while the spatial resolution of the training images was about 0.2 mm per pixel.

### Comparison with other methods for panicle counting

Panicle-SEG (Xiong et al., 2017) was an algorithm for rice panicle segmentation. Combining Panicle-SEG with an appropriate image processing method, the number of the panicles in the image can be obtained by counting the connected components. For the binary images obtained by Panicle-SEG, opening and closing operations with a 5 × 5-size kernel was performed to remove noise and separate occluded rice panicles. Then the number and area of connected components were obtained. In order to deal with the occluded rice panicles, the median area of the connected components in each image was calculated. If the area of a component was larger than twice of the median area, the component’s area would be divided by the median area and then round up to an integer, which was regarded as the number of rice panicles corresponding to the component. For other connected components, each one was regarded as one rice panicle. The panicle number for each image was then computed.

MHW-PD (Xu et al., 2020) was an advanced algorithm for rice panicle count and was similar to the proposed method. In comparison, this paper calculated the mean counting accuracy in their manner (Xu et al., 2020), which is shown in Eq. (11).


(11) $$P_{c}=\frac{N_{c\,o\,r}}{N_{r\,e\,a\,l}}\times 100\left(\%\right)$$

where P_*c*_ is the counting accuracy, N_*cor*_ is the correct (true positive) number of rice panicles detected by the model and N_*real*_ is the actual number of the rice panicles in the test set.

## Results and discussion

**FIGURE 6:**
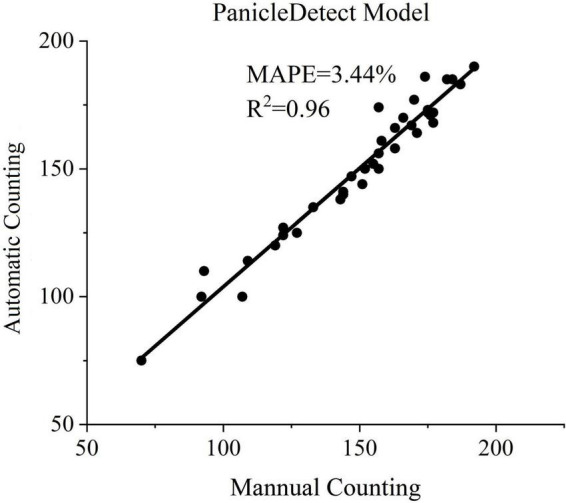
Performance of the PanicleDetect model.

We tested the proposed panicle detection algorithm using 37 field rice images. Each rice image belonged to a different accession. Convolution neural network, YOLOv5x was trained for panicle detection. The results of YOLOv5x are shown in Figure 6. The mean values of the MAPE, Precision, Recall, F-measure, R^2^ and Accuracy were 3.44%, 96.24%, 95.81%, 95.98%, 0.96, and 92.77%, respectively.

### Comparison of different object detection models for panicle counting

Four object detection models: YOLOv3 (Redmon and Farhadi, 2018), YOLOv5l, YOLOv5x and Faster R-CNN (Ren et al., 2017) were tested and compared for panicle counting (Table 1). The average time consumption for detecting one sub-image was also computed. The detection was run on the same environment as training.

**TABLE 1:** Comparison of different object detectors for panicle counting.

Object detector	MAPE	Precision	Recall	F1-measure	R^2^	Accuracy	Time/ms
YOLOv3	4.18%	96.54%	93.13%	94.77%	0.96	90.80%	103.72
YOLOv5l	3.99%	97.00%	95.12%	95.99%	0.94	92.82%	83.61
YOLOv5x	3.44%	96.24%	95.81%	95.98%	0.96	92.77%	92.58
Faster R-CNN	3.37%	96.31%	96.01%	96.12%	0.95	92.92%	159.47

The results showed that the proposed counting method had good adaptability to different object detection networks. Furthermore, YOLOv5x and Faster R-CNN outperformed the other two networks. Considering the detection efficiency, YOLOv5x was selected as the optimal network for the PanicleDetect algorithm.

### Comparison of different methods for removing repeated detections in the adjacent sub-images

**FIGURE 7:**
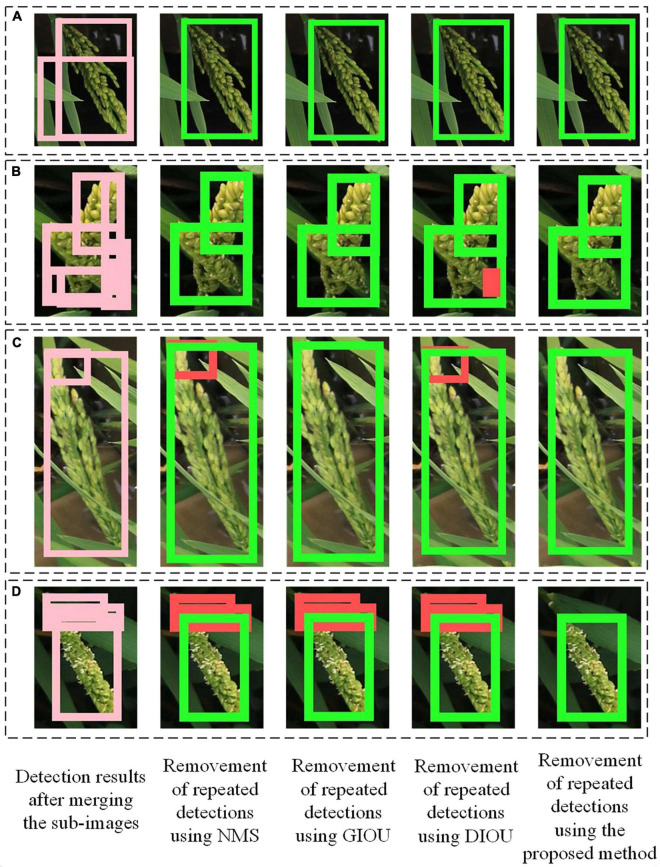
Detection results of rice image processed by the proposed method and other methods. **(A)** The areas of the overlapping boxes were similar. **(B–D)** The areas of the overlapping boxes varied greatly. In the detection results, green boxes are correct bounding boxes and red boxes are FP boxes.

The proposed method using IOB and BOU was compared with the NMS IOU, GIOU and DIOU methods for removing the repeated detections. Six indicators were used to evaluate the results. The results are shown in Table 2. The results showed that the overall performance of the proposed method was better than other methods. The main reason for why the proposed method outperformed the other methods is presented in Figure 7. When the areas of the overlapping boxes were similar, high overlapping degree of the boxes would lead to high IOU, GIOU, DIOU, IOB and BOU, so all the 4 methods can remove the repeated boxes correctly (Figure 7A). However, when the areas of the overlapping boxes varied greatly, even if the overlapping degree of the boxes were very high, for instance, one box completely covered the other box, the IOU, GIOU and DIOU would be also low. In contrast, the IOB and BOU would still be high. In this case, the repeated boxes might be kept by the other methods, but be removed by the proposed method (Figures 7B–D). Thus, the proposed method used IOB and BOU performed better than the other methods.

**TABLE 2:** Evaluation of detection results of different methods.

	MAPE	Precision	Recall	F1-measure	R^2^	Accuracy
Proposed method	3.44%	96.24%	95.81%	95.98%	0.96	92.77%
NMS IOU	3.76%	95.92%	95.03%	95.40%	0.93	91.78%
GIOU	3.99%	95.77%	95.75%	95.69%	0.93	92.23%
DIOU	4.21%	93.22%	94.84%	93.95%	0.94	89.30%

### Detection results under different rice accessions and illumination environments

**FIGURE 8:**
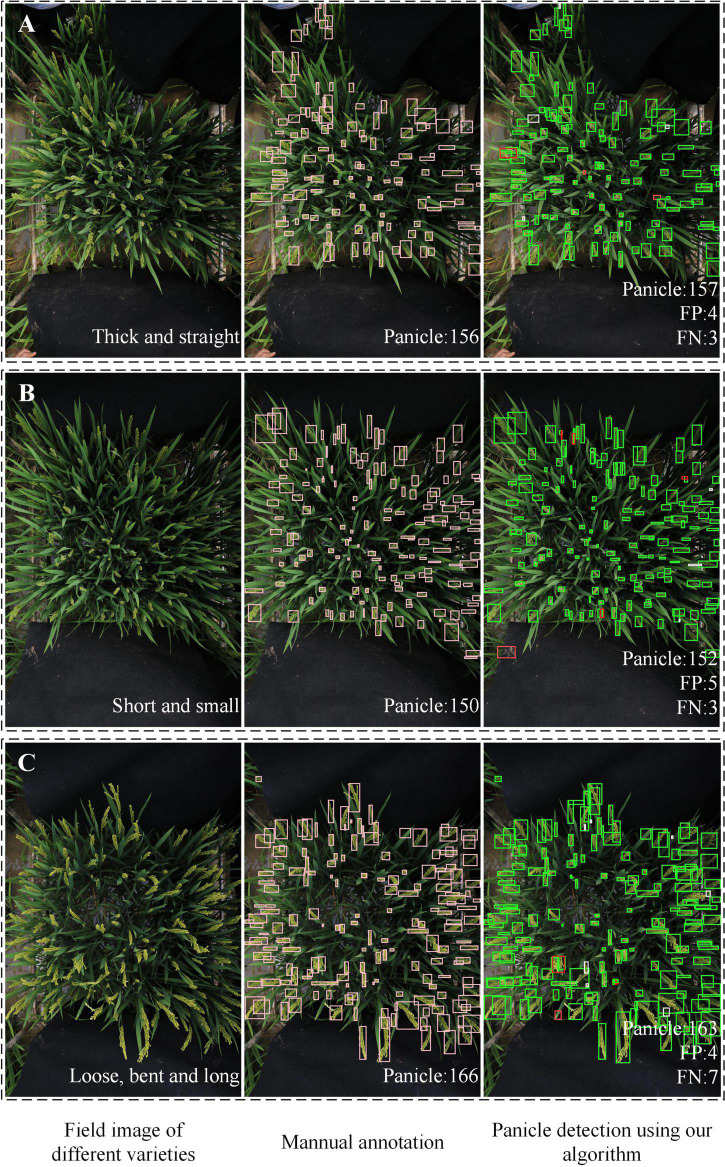
Examples of rice field images with different rice accessions. **(A)** Thick and straight. **(B)** Short and small. **(C)** Loose, bent and long. In the detection results, green boxes are correct bounding boxes; white boxes are FN boxes; red boxes are FP boxes.

The appearance of rice panicles varies greatly among different rice accessions. For example, the panicles were thick and straight in Figure 8A short and small in Figure 8B, and loose, bent and long in Figure 8C. In addition, the large growth density would cause occlusion between panicles or between leaves and panicles. Results showed that the proposed method was robust for detecting panicles of different rice accessions.

**FIGURE 9:**
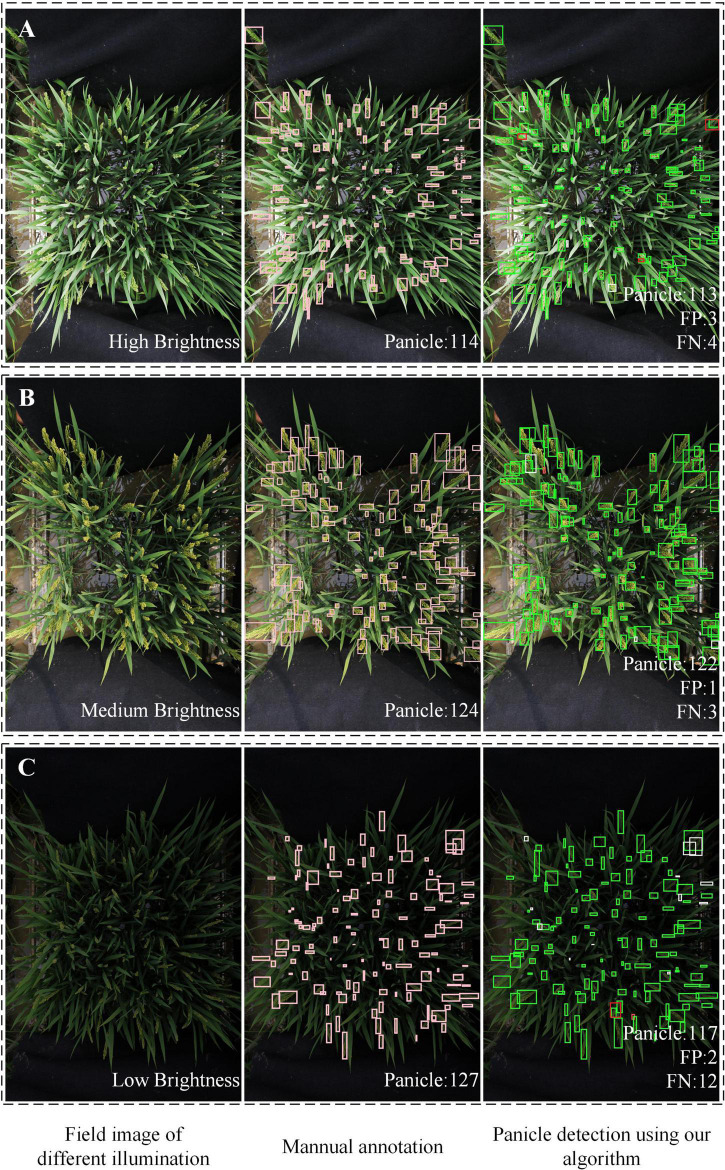
Examples of rice field images with different illumination. **(A)** High brightness. **(B)** Medium brightness**. (C)** Low brightness. In the detection results, green boxes are correct bounding boxes; white boxes are FN boxes; red boxes are FP boxes.

The illumination of the different images varied due to the outdoor environment. Therefore, it was important for the model to accurately detect images under different illumination conditions. As illustrated in Figure 9, for the high brightness image (Figure 9A), the FP is 3 and the FN is 4. In the image of medium brightness (Figure 9B), the FP was 1 and the FN was 3. In the image of low brightness (Figure 9C), the FP was 2 and the FN was 12. From the results, the model performed best for medium brightness image detection. And the model was robust to the images with different brightness. Specially, for images with extremely low brightness, such as Figure 9C, manual annotation was error-prone, labor-intensive and inefficient. However, the proposed algorithm performed well.

### Panicle detection of images with different image size and spatial resolution

Different devices and methods are used for capturing rice field images, which may cause differences in spatial resolution. Therefore, it is important that the proposed counting algorithm is robust for the images with different spatial resolution. Table 3 shows the performance of the PanicleDetect model with different input size/spatial resolution. The results showed that, enlarging or reducing the size of the input image by nearly twice, the MAPE of the model detection results could be kept within 5%, meaning that the proposed algorithm was robust to different spatial resolution.

**TABLE 3:** Comparison of the different input size/spatial resolution for the PanicleDetect model.

Input image size	MAPE	Precision	Recall	F1-measure	R^2^	Accuracy
256 × 256	4.60%	94.24%	94.94%	94.51%	0.93	90.21%
416 × 416	3.44%	96.24%	95.81%	95.98%	0.96	92.77%
608 × 608	3.64%	96.71%	95.54%	96.06%	0.95	92.87%
800 × 800	3.11%	97.08%	95.99%	96.48%	0.96	93.62%
1056 × 1056	3.50%	95.88%	95.87%	95.82%	0.95	92.47%

### Panicle detection of the field images taken by unmanned aerial vehicle

**FIGURE 10:**
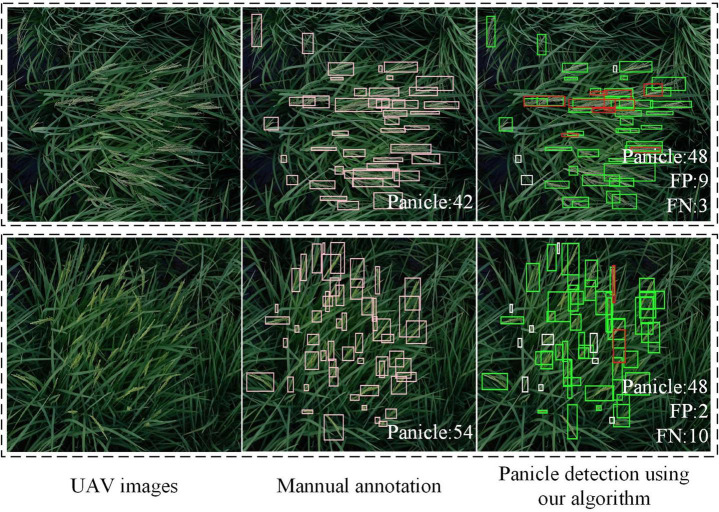
Detection results of field images taken by UAV. In the detection results, green boxes are correct bounding boxes; white boxes are FN boxes; red boxes are FP boxes.

Using UAV to capture the rice field images is convenient and efficient. Thus, it is meaningful that the proposed algorithm can detect the panicles accurately in the field images taken by UAV. But the images taken by UAV may have the problems of low-resolution or defocus blur, which will bring challenges to rice panicle detection. The PanicleDetect model was trained using data augmentation with image blur, so the model was more robust for this situation. Figure 10 shows the detection results of two representative images with about 1000 × 1000 pixels taken by UAV. The results showed that, the proposed algorithm had a relatively high accuracy for counting panicles in UAV images.

### Comparison with other methods for panicle counting

Panicle-SEG (Xiong et al., 2017) was an algorithm for rice panicle segmentation. Combining Panicle-SEG with an appropriate image processing method, the number of the panicles in the image can be obtained by counting the connected components. The counting method was described in detail in the method section. The same testing set was used to evaluate the performance of panicle counting using segmentation method, and the mean values of the MAPE, Precision, Recall, F-measure, R^2^ and Accuracy were 13.59%, 79.39%, 80.66%, 79.49%, 0.68, and 71.79%, respectively. This counting method has difficulty in dealing with the occluded rice panicles and rice panicles with different sizes. Therefore, the accuracy of this method was relatively low.

MHW-PD (Xu et al., 2020) was an advanced algorithm for rice panicle count and was similar to the proposed method. Specifically, this algorithm firstly cut the images into sub-images without overlapping, then detected the panicles in the sub-images using Faster R-CNN and fused the results. The panicle count accuracy of MHW-PD achieved about 93% for images with 0∼30 panicles per image, and about 87% for images with 31∼80 panicles per image. In comparison, this paper calculated the mean counting accuracy in their manner. The results showed that the proposed method reached an accuracy of 95.81% for images with 75∼190 panicles per image. In conclusion, the proposed method is able to process images with much higher number of rice panicles, and can maintain a higher accuracy.

## Conclusion

It is challenging and meaningful to accurately measure panicle number in the field. This paper proposed a rice panicle counting algorithm that are especially designed for field images with extremely large image size. Instead of greatly resizing or cutting images without overlapping, small panicles can be preserved intact in the images. This algorithm enables the object detect networks, which were designed for input of relatively small image size, to detect small objects in large images. For field images of 6000 × 4000 pixels with an average of 140 panicles per image, the MAPE of this algorithm was 3.44%. The proposed method was proved to be robust and accurate for counting panicle in field rice images of different illumination, rice accessions, and spatial resolution. The proposed method also performed well on UAV images. One limitation for this work is that the proposed method was only tested at a single planting density, which was slightly higher than the typical planting density used for rice cultivation. Panicle detection at different planting densities will be tested in our future work. Generally, the method was robust and especially useful for panicle detection of extremely large images. In addition, this algorithm can be visited online so the researchers can use the algorithm to get panicle numbers conveniently.

## Data availability statement

The raw data supporting the conclusions of this article will be made available by the authors, without undue reservation.

## Author contributions

XW and LD developed the detection method, conducted the experiments, and wrote the manuscript. QL, WY, CH, and XL were assistant to the experiments. GC and LX contributed in rice materials and rice managements. All authors read and approved the final manuscript.

## Footnotes

1
http://44g29257r5.zicp.vip/

## Supplementary material

Supplementary Video 1 The instruction of the website for rice panicle counting.

## Abbreviations

CNN: convolutional neural network
UAV: unmanned aerial vehicle
TP: true positive
TN: true negative
FP: false positive
FN: false negative
NMS: non-maximum suppression
IOU: intersection over union.
