# Supplementary material for: Field rice panicle detection and counting based on deep learning
Source: Front Plant Sci. 2022 Aug 12;13:966495. doi: 10.3389/fpls.2022.966495 (PMC9416702; doi:10.3389/fpls.2022.966495)
Supplement: Supplementary file 2 [file Table_1.DOCX]

Table 1 Rice accessions used in the study

| Name of accession | Name of accession | Name of accession |
| --- | --- | --- |
| HuaiDao5 | Wu9516 | SiDao12 |
| ShengWuNuo0146 | NanNong6427 | SiDao785 |
| ShengDao14 | ZhenDao523 | SiDao15 |
| ShengXiang145 | Yang95-38 | Si26 |
| ShengDao285 | Huai9508 | Si301 |
| ZhenDao9424 | WuYuJing3 | Si378 |
| JinDao253 | HuaiDao7 | Si2333 |
| YangGuang200 | LiShuiNongHuZhongJingXuan | YanJing10 |
| LinDao18 | Wu2817 | YanJing11 |
| LinDao15 | SiDao0961 | YangYuJing2 |
| LinDao17 | SiDao09-30 | YanJing16 |
| LinDao9 | YanDao9 | YanDao8 |
| YangGuang800 | Ning5046 | YanDao10 |
| RunNong4 | ChangJing09-3 | YanDao11 |
| JiDao1 | WuYunJing24 | YanDao13293 |
| JiDao3 | ChangShu-1 | YanDao5228 |
| JiDao4 | Wu910 | TaiJing4366 |
| JiDao5 | ChangJing10-6 | TaiJing50565 |
| JiDao6 | ChangJing10-9 | YangJing4038 |
| JiDao8 | NingJing4 | YangJing4227 |
| JiDao9 | NingJing5 | YangJing805 |
| LuZiDao5 | NingJing7 | YangJing282 |
| LuZiDao6 | W3668 | YangJing103 |
| DaoPin15 | XuHan1 | YangJing113 |
| DaoPin38 | Xu32646 | YangJing306 |
| DaoPin39 | Xu36618 | NanJing47 |
| DaoPin56 | LianJing9 | NanJing49 |
| YangNuo5 | LianJing11 | NanJing51 |
| YangNuo204 | LianJing12 | NanJing52 |
| Huai9306 | ZhongDao1 | NanJing53014 |
| ZhenXiang24 | ZhongZuoDao2 | NanJing53015 |
| Yang92-133 | LianJing14ZJ29 | ZhenDao14 |
| LianJing8410 | LianJing13228 | ZhenDao15 |
| Huai87089 | ZhongZuo13264 | ZhenDao9469 |
| ZhenDao5262 | LianJing14JD24 |  |
